# CircZDBF2 up-regulates RNF145 by ceRNA model and recruits CEBPB to accelerate oral squamous cell carcinoma progression via NFκB signaling pathway

**DOI:** 10.1186/s12967-022-03347-1

**Published:** 2022-04-01

**Authors:** Liang Rong, Bo Chen, Ke Liu, Bingyao Liu, Xinyao He, Juan Liu, Junxia Li, Maodian He, Lei Zhu, Ke Liu, Xiaolei Shi, Yi Shuai, Lei Jin

**Affiliations:** 1 Department of Stomatology, Affiliated Jinling Hospital, Medical School of Nanjing University, Nanjing, 210002 Jiangsu China; 2 Department of Stomatology, Jinling Hospital, Nanjing Medical University, Nanjing, 210002 Jiangsu China; 3 Department of Endodontics, The Affiliated Stomatological Hospital of Nanjing Medical University, Nanjing, 210002 Jiangsu China; 4 Department of Stomatology, Jinling Hospital, School of Stomatology, Southern Medical University, Guangzhou, 510515 Guangdong China

**Keywords:** OSCC, circZDBF2, RNF145, miR-362-5p, miR-500b-5p, CEBPB

## Abstract

**Background:**

Oral squamous cell carcinoma (OSCC), as one of the commonest malignancies showing poor prognosis, has been increasingly suggested to be modulated by circular RNAs (circRNAs). Through GEO (Gene Expression Omnibus) database, a circRNA derived from ZDBF2 (circZDBF2) was uncovered to be with high expression in OSCC tissues, while how it may function in OSCC remains unclear.

**Methods:**

CircZDBF2 expression was firstly verified in OSCC cells via qRT-PCR. CCK-8, along with colony formation, wound healing, transwell and western blot assays was performed to assess the malignant cell behaviors in OSCC cells. Further, RNA pull down assay, RIP assay, as well as luciferase reporter assay was performed to testify the interaction between circZDBF2 and RNAs.

**Results:**

CircZDBF2 expressed at a high level in OSCC cells and it accelerated OSCC cell proliferation, migration, invasion as well as EMT (epithelial-mesenchymal transition) process. Further, circZDBF2 sponged miR-362-5p and miR-500b-5p in OSCC cells to release their target ring finger protein 145 (RNF145). RNF145 expressed at a high level in OSCC cells and circZDBF2 facilitated RNF145 transcription by recruiting the transcription factor CCAAT enhancer binding protein beta (CEBPB). Moreover, RNF145 activated NFκB (nuclear factor kappa B) signaling pathway and regulated IL-8 (C-X-C motif chemokine ligand 8) transcription.

**Conclusion:**

CircZDBF2 up-regulated RNF145 expression by sponging miR-362-5p and miR-500b-5p and recruiting CEBPB, thereby promoting OSCC progression via NFκB signaling pathway. The findings recommend circZDBF2 as a probable therapeutic target for OSCC.

**Graphical Abstract:**

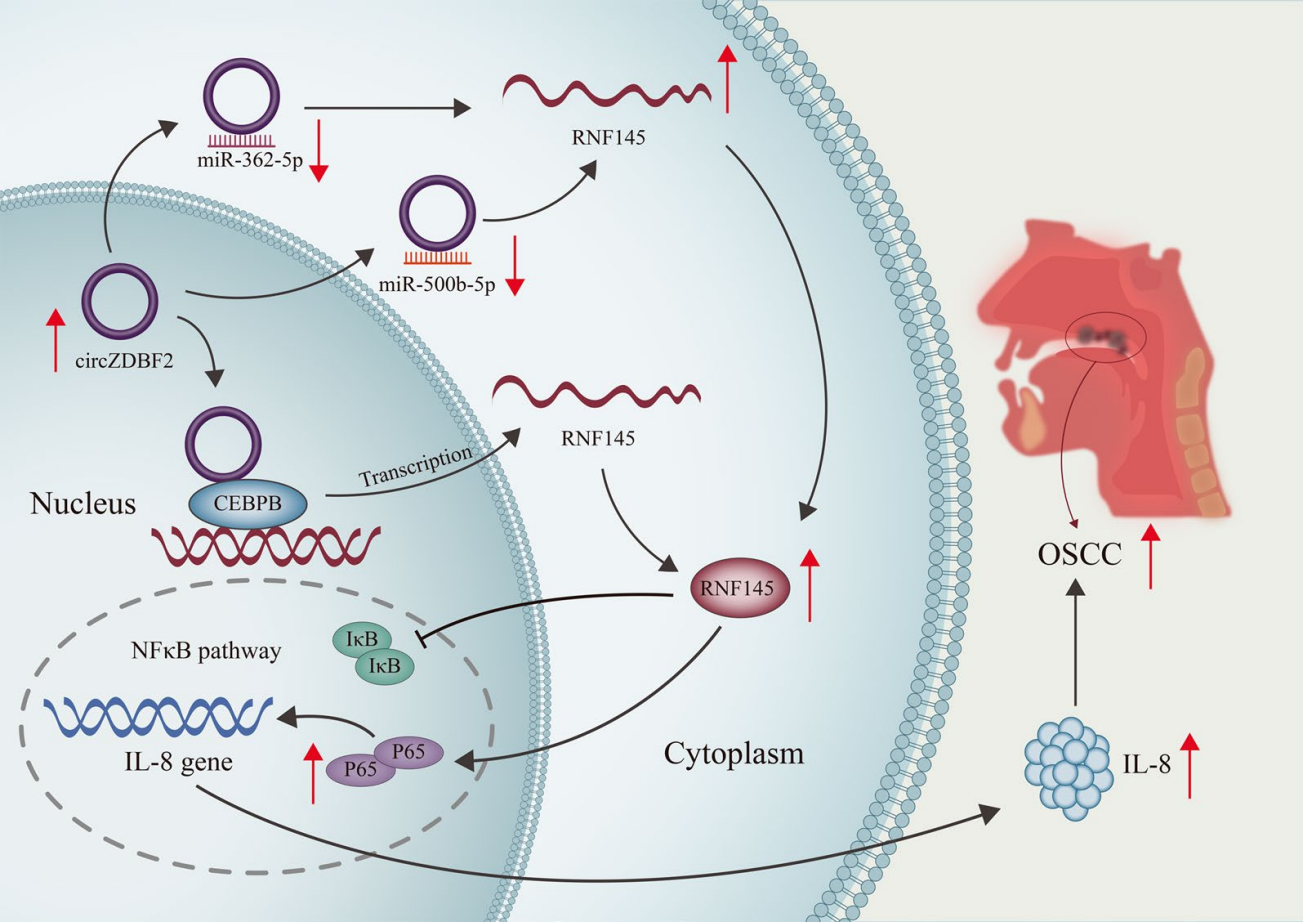

**Supplementary Information:**

The online version contains supplementary material available at 10.1186/s12967-022-03347-1.

## Background

As one of the commonest form of oral cancer, oral squamous cell carcinoma (OSCC) ranks the sixth largest cancer having a high mortality rate around the world [1, 2]. At present, there are more than 300,000 new cases of OSCC patients in the world every year [3]. Main treatment strategies of OSCC are surgery with adjuvant radiation or chemoradiation [4, 5]. In spite of the progress taken in OSCC treatment, the five-year survival rate is still very low. Some patients at advanced stage will have tumor recurrence and distant metastasis, so the prognosis of patients is poor [6]. Investigating the pathogenesis of OSCC and exploring new therapeutic targets are hence necessary measures to improve this situation.

Circular RNAs (circRNAs) are considered as a special class of non-coding RNAs (ncRNAs) which are formed from exons or introns via special selective shearing [7]. They are single-stranded covalently closed circular transcripts lacking 5′ caps and 3′ poly(A) tails, which enables them with higher capacity to stand up to environmental degradation [8]. In recent years, accumulating researches have confirmed the involvement of circRNAs in various biological processes of cancer cells such as cell proliferation and cell apoptosis [9]. The dysregulation of circRNAs has been identified in many cancer types in which they promote or inhibit cells [10]. For example, circ-RanGAP1 regulates vascular endothelial growth factor A (VEGFA) expression through miR-877-3p so as to aggravate cell invasion and metastasis in gastric cancer [11]. Knockdown of circ-CPA4 inhibits cell growth and facilitates cell death in non-small cell lung cancer cells by inhibiting PD-L1 (programmed cell death 1 ligand 1) through serving as a RNA sponge for let-7 [12]. Circ-SMARCA5 exerts a suppressive impact on colorectal cancer malignancy via sequestering miR-39-3p to elevate AT-rich interaction domain 4B (ARID4B) [12]. Also, it is reported that circ_0000140 attenuates OSCC cell growth via miR-31 so as to inhibit Hippo signaling pathway [13]. According to GEO database (GSE145608), hsa_circ_0002141, a circRNA originated from zinc finger DBF-type containing 2 (ZDBF2) (namely circZDBF2), was discovered to be with high expression in OSCC tissues, while it has not been investigated in cancer developmental present, let alone in OSCC.

Accumulating studies have indicated that circRNAs can exert regulatory functions in cancer development through interaction with microRNAs (miRNAs) [14]. MiRNAs are short RNA molecules which are 18–22 nucleotides in size [15]. They exert regulatory influence in the process of human cancers via repressing target messenger RNA (mRNA) translation or accelerating mRNA degradation [16]. For example, miR-106b-5p promotes the lung metastasis of breast cancer by decreasing Calponin 1 (CNN1) [17]. MiR-944 inhibits colorectal cancer cell migration as well as invasion by targeting MACC1 (MET transcriptional regulator MACC1) [18]. MiR-133a-3p inhibits collagen type I alpha 1 chain (COL1A1) to suppress OSCC malignant cell behaviors [19].

In this research, we were intended to probe into the specific role of circZDBF2 in OSCC cells and the molecular mechanism of circZDBF2 with RNAs.

## Methods

### Cell culture and treatment

Among three OSCC cell lines (SCC9, SCC15 and SCC25), SCC9 and SCC15 cells were obtained from American Type Culture Collection (ATCC, Manassas, VA, USA) while SCC25 cells were from Procell Life Science & Technology Co., Ltd. (Wuhan, China). A healthy human oral keratinocyte (HOK) cell line was purchased from Binsui Biotechnology Co., Ltd. (Shanghai, China). All the cell lines were cultivated in Dulbecco's Modified Eagle Medium (DMEM) (Gibco, Gaithersburg, MD, USA) and supplemented with 10% fetal bovine serum (FBS) (Gibco, USA) and 100 U/ml penicillin/streptomycin under 5% CO_2_ at 37 °C. For circRNA characterization, 3 U/mg of RNase R from Epicentre Technologies (Madison, WI) and 2 mg/ml of Actinomycin D (Act D) from Sigma-Aldrich (St. Louis, MO) were used.

### Cell transfection

To knock down circZDBF2 or RNF145 expression, short hairpin RNAs (shRNAs) specifically targeting circZDBF2 (sh-circZDBF2-1/2/3) or RNF145 (sh-RNF145-1/2/3), together with the negative control (NC) were synthesized from GenePharma (Shanghai, China). In addition, pcDNA3.1/circZDBF2, pcDNA3.1/RNF145 and pcDNA3.1/CEBPB, along with their negative control pcDNA3.1 obtained from GenePharma were constructed respectively for the overexpression of genes. The miR-362-5p mimics/inhibitor, miR-500b-5p mimics/inhibitor and their negative controls (NCs) (mimics-NC/inhibitor-NC) were designed via Ribobio (Guangzhou, China). Cell transfection was taken using Lipofectamine 2000 (Invitrogen Corp., Carlsbad, CA, USA). Forty-eight hours later, cells were collected for further investigations.

### Quantitative real-time PCR (qRT-PCR)

Total RNA was extracted in SCC9 and SCC15 cells by the application of TRIzol® reagent (Takara, Japan) in line with the protocols of supplier. Prime Script™ RT Master Mixture (Takara 11141ES10, Japan) or TaqMan® MicroRNA RT kit (4366596, Applied Biosystems™, Foster City, CA, USA) was utilized for RNA reverse transcription (RT). Then, qPCR was implemented with the qRT-PCR Kit (QR0100-1KT, Sigma-Aldrich, USA) by using StepOnePlus™ Real-time PCR Systems (Applied Biosystems™). Relative expression levels were calculated using the 2^−ΔΔCt^ method, with U6 small nuclear RNA (U6) as the endogenous control to normalize miRNA expression and glyceraldehyde-3-phosphate dehydrogenase (GAPDH) as the endogenous control to normalize mRNA/circRNA expression. Related primer sequences were exhibited in Additional file 8: Table S1.

### Cell counting kit-8 (CCK-8) assay

Transfected SCC9 and SCC15 cells harvested at the logarithmic growth phase were incubated into 96-well plates (5 × 10^3^ cells/well) with complete culture medium at 37 ℃ for 24, 48, 72 h. Then, cells were cultivated with 10 μL of CCK-8 reagent (ab228554, Abcam, UK) for 1 h. The absorbance at 450 nm was assayed by microplate reader.

### Colony formation assay

Transfected SCC9 and SCC15 cells were prepared in 6-well plates (800 cell/well) for 14 days of colony formation. The culture medium was discarded and the cells were washed with phosphate buffer saline (PBS) for two times. The colonies were fixed with methanol and dyed by 0.5% crystal violet solution. Finally, colonies with no less than 50 cells were counted manually.

### Transwell-invasion assays

Serum-free medium containing transfected SCC9 and SCC15 cells was planted on the top of 24-well transwell chambers (5 × 10^3^ cells/well) along with Matrigel, and the lower chambers were loaded with complete medium. Twenty-four hours later, the medium was discarded and non-invaded cells were removed by a cotton swab. The bottom of the chamber was fixed by methanol solution for 15 min and dyed with crystal violet for 10 min. The cells that had invaded were counted at 5 randomly chosen visual fields under a microscope (Smart zoom 5, Zeiss).

### Wound healing assay

Cell samples were seeded in 6-well plates (1 × 10^6^ cells/well) for reaching 100% cell confluence, and then treated with 200-μL pipette tip. The scratched cells were removed, the serum-free medium was added, and then the cells were cultivated in an incubator at 37 °C with 5% CO_2_. The width of 3–5 randomly selected spots at 24 h was recorded and the distance of wound closure was analyzed.

### Western blot

Total protein extracted from SCC9 and SCC15 cells was isolated by Radio Immunoprecipitation Assay (RIPA) lysis buffer and then subjected to isolation through electrophoresis using sodium dodecyl sulfate polyacrylamide gel electrophoresis (SDS-PAGE;10%). The samples transferring on polyvinylidene fluoride (PVDF) membranes were blocked via 5% nonfat milk and then subjected to incubation with primary antibodies against ZO-1 (ab276131, Abcam, UK), E-cadherin (ab40772, Abcam, UK), N-cadherin (ab76011, Abcam, UK), Vimentin (ab92547, Abcam, UK), p50 (ab32360, Abcam, UK), p65 (ab16502, Abcam, UK), IκBɑ (#9242, Cell Signaling Technology, China), and GAPDH (China Kangcheng Biotechnology Co., Ltd.) as the internal control. After being rinsed in Tris-buffered saline-tween (TBST), samples were incubated with secondary antibody labeled with horseradish peroxidase (HRP) and finally exposed to electrochemiluminescent (ECL) luminescent liquid (Santa Cruz Biotechnology, Santa Cruz).

### Fluorescence in situ hybridization (FISH) assay

The circZDBF2-specific RNA FISH probe procured from Ribobio was used for cellular analysis as instructed by provider. The fixed cell samples were rinsed in PBS, and then air-dried, followed by the hybridization with FISH probe. Cell nuclei were stained using 4',6-diamidino-2-phenylindole (DAPI) solution purchased from Beyotime (Shanghai, China), and the fluorescent images were captured by GLomax20/20 fluorescence microscope (Promega).

### RNA immunoprecipitation (RIP) analysis

RIP analysis was made via Magna RIP™ RNA-Binding Protein Immunoprecipitation Kit (Millipore, Bedford, MA) based on user guides. SCC9 and SCC15 cells were lysed by RIP buffer and cell lysates were collected. Magnetic beads were conjugated with argonaute2 antibody (anti-AGO2; ab186733, Abcam, UK) or immunoglobulin G antibody (anti-IgG; ab205718, Abcam, UK). After rinsing, the precipitated RNA went through qRT-PCR analysis.

### Chromatin immunoprecipitation (ChIP) assay

ChIP was conducted utilizing EZ-ChIP chromatin immunoprecipitation kit (Millipore, Bedford, USA) in accordance with user guides. SCC9 and SCC15 cells were cross-linked in 4% paraformaldehyde, sonicated into chromatin fragments of 200–1000-bp and incubated with the antibodies against CEBPB (ab264305, Abcam, UK), with Anti-IgG served as the negative control. The specific primers for the RNF145 promoter were used, and precipitated DNA was detected by qRT-PCR.

### RNA pull down assay

Based on the protocols of supplier, we utilized the Pierce Magnetic RNA–Protein Pull-Down Kit (Thermo Fisher Scientific, Waltham, MA) to perform this assay. Biotin-labeled circZDBF2 (Bio-circZDBF2) or RNF145 (Bio-RNF1452) probes were incubated with cell extracts and streptavidin magnetic beads, with Bio-NC as the negative control. Finally, the RNA complexes bound to beads were analyzed by qRT-PCR. In this study, three experimental groups (Bio-NC, Bio-circZDBF2/RNF145-WT and Bio-circZDBF2/RNF145-Mut) were constructed respectively to evaluate the binding capacity between circZDBF2/RNF145 and miR-362-5p/miR-500b-5p.

### Dual-luciferase reporter analyses

The wild-type (WT) and mutant (Mut) miR-362-5p or miR-500b-5p binding sites within circZDBF2 sequence or RNF145 3′UTR (3′ untranslated region) were sub-cloned into pmirGLO dual-luciferase vector, and pmirGLO-circZDBF2-WT/Mut or pmirGLO-RNF145 3’UTR-WT-Mut plasmids were hence constructed. The pmirGLO plasmids were co-transfected with miR-362-5p mimics or miR-500b-5p mimics and their NC mimics in SCC9 and SCC15 cells. Forty-eight hours later, the luciferase activity was estimated with Dual-Luciferase Reporter Assay System (Promega Corporation, Fitchburg, WI).

### In vivo tumor growth assay

Total of 2 × 10^6^ SCC9 cells transfected with sh-NC or sh-circZDBF2-1 were injected into the male BALB/c nude mice (6–8-week old; total number = 8 mice; n = 4 mice/group; Slac Laboratories, Shanghai, China). The animal assay was performed strictly in line with the protocol approved by the Ethical Committee of Affiliated Jinling Hospital, Medical School of Nanjing University, with the approval numbered 2020DZGZRZX-078. Tumor volume was calculated every 3 days following the formula: volume = length × width^2^/2. Four weeks later, mice were sacrificed after which tumors were excised and weighed.

### Immunohistochemistry (IHC)

Paraffin-embedded tissues from in vivo tumor growth assay were first fixed by 4% paraformaldehyde, embedded in paraffin and cut into consecutive sections at 4-μm thick for IHC assay. Then sections were incubated with anti-Ki67 (ab15580, Abcam, UK) at 4 °C overnight, and then with secondary antibody for 30 min. Images were taken using an Olympus microscope (Olympus Corporation, Japan).

### Statistical analysis

Each experiment was performed for three times in this research. Statistical analysis was made via SPSS 23.0. Mean ± standard deviation (SD) was used to show the results. After validating normal distribution via shapiro test and homogeneity of variance via Levene test, data were analyzed via Student’s *t*-test or one-way analysis of variance (ANOVA) for difference analyses. *P* < 0.05 was of great importance for statistically significant difference.

## Results

### CircZDBF2 is considerably overexpressed in OSCC cells

**Fig. 1:**
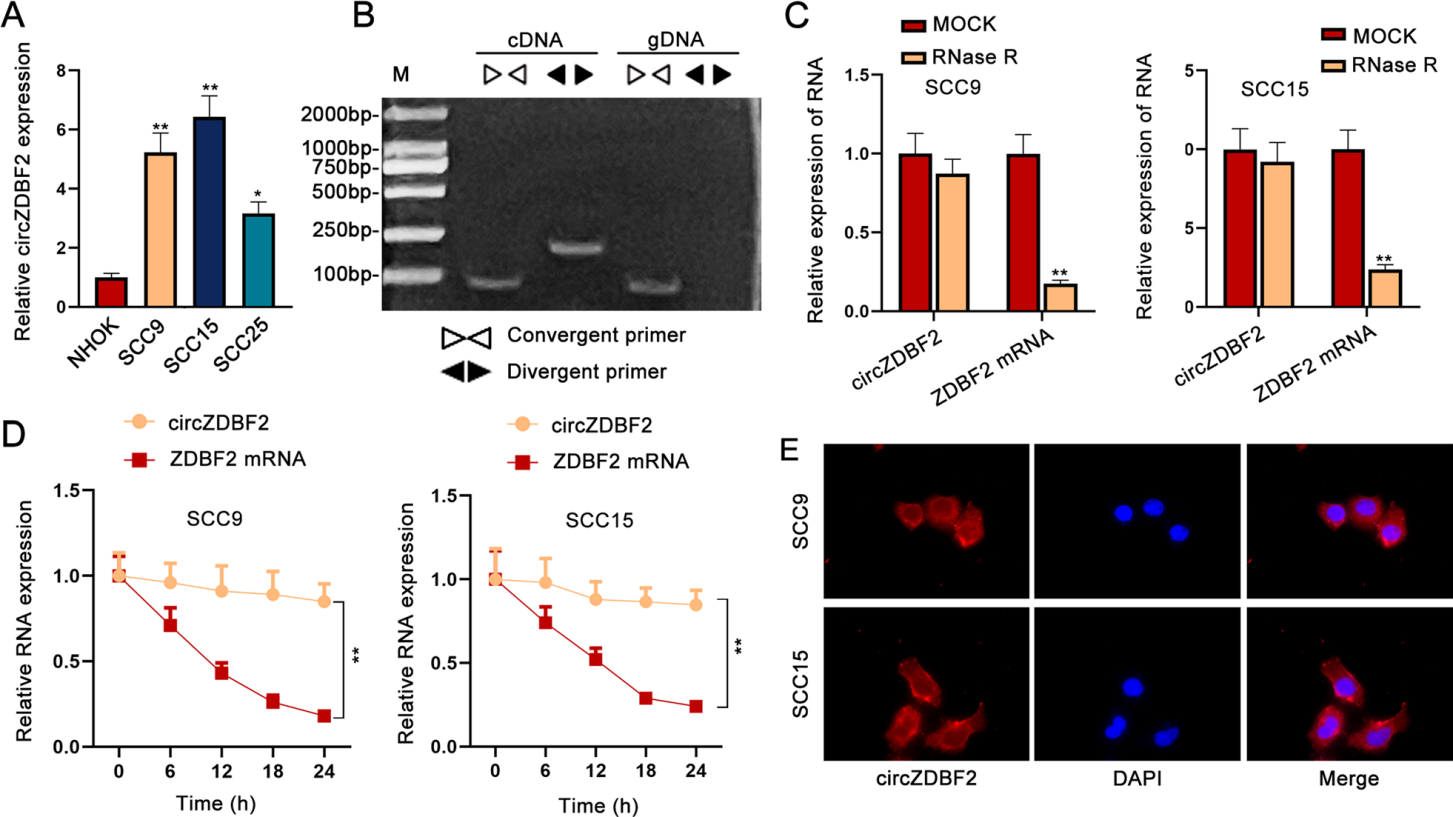
CircZDBF2 is considerably overexpressed in OSCC cells. **A** CircZDBF2 expression in OSCC cell lines (SCC9, SCC15, SCC25) and HOK cell line was detected by qRT-PCR analysis. **B** PCR-agarose gel electrophoresis (AGE) was taken to verify the cyclization of circZDBF2. **C** ZDBF2 mRNA level and circZDBF2 expression in OSCC cells was measured by qRT-PCR upon RNase R treatment. **D** qRT-PCR was utilized to measure the stability of circZDBF2 and in OSCC cells treated with Act D. **E** The subcellular location of circZDBF2 in SCC9 and SCC15 cells was identified by FISH assay. ^*^P < 0.05, ^**^P < 0.01

First of all, we discovered that hsa_circRNA_002141 (namely hsa_circ_0002141, here called circZDBF2) was highly expressed in oral cancer cell lines (HSC3, Sa3 and SAS) compared to human normal oral keratinocytes (HNOKs) according to GSE145608 (Additional file 1: Fig. S1A). The discovery interested us to probe into the probable role of circZBF2 in OSCC. Before that, circZDBF2 expression was assessed in OSCC cell lines (SCC9, SCC15, SCC25) and a healthy human oral keratinocyte (HOK) cell line. Results from qRT-PCR showed that circZDBF2 was overexpressed in OSCC cells (especially in SCC9 and SCC15 cells) compared with normal cells (Fig. 1A). Convergent primers were constructed to amplify linear ZDBF2 and divergent primers to amplify circZDBF2, with cDNA (complementary DNA) and gDNA (genomic DNA) extracted from cells as templates. It was shown that circZDBF2 was amplified from cDNA only by divergent primers (Fig. 1B). Further, unlike linear ZDBF2 mRNA, circZDBF2 showed no evident change in OSCC cells after being treated with RNase R (Fig. 1C), suggesting the circular structure of circZDBF2. Moreover, we added Act D (a transcription inhibitor) in SCC9 and SCC15 cells and found that circZDBF2 was more stable than liner ZDBF2 (Fig. 1D). These results verified the cyclization of circZDBF2. We further analyzed the cellular distribution of circZDBF2 by FISH assay, which indicated that circZDBF2 existed in the nucleus as well as the cytoplasm of OSCC cells (Fig. 1E). To sum up, circZDBF2 with a loop structure is with high expression in OSCC cells.

### CircZDBF2 accelerates cell proliferation, invasion, migration and EMT process in OSCC

**Fig. 2:**
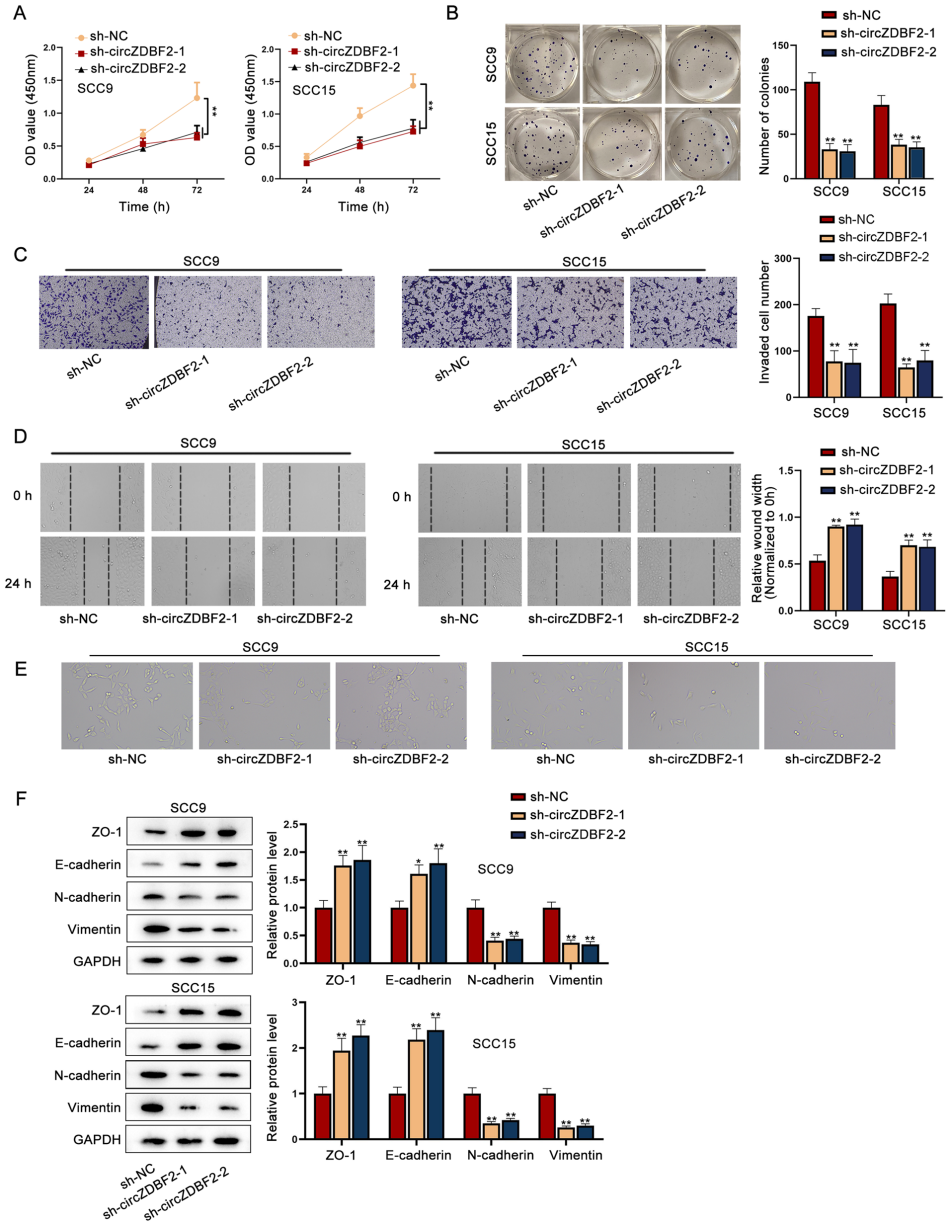
CircZDBF2 accelerates cell proliferation, invasion, migration and EMT process in OSCC. **A**, **B** CCK-8 as well as colony formation assay was employed to measure cell viability and proliferation when circZDBF2 was silenced in OSCC cells. **C**, **D** Transwell assay, together with wound healing assay was performed to measure cell invasion and migration when circZDBF2 was inhibited in OSCC cells. **E**, **F** EMT phenotype in SCC9 and SCC15 cells transfected with sh-circZDBF2 was detected. ^*^P < 0.05, ^**^P < 0.01

By transfecting sh-circZDBF2-1/2/3 plasmids into SCC9 and SCC15 cells, we obtained the reduced circZDBF2 expression (Additional file 1: Fig. S1B), and sh-circZDBF2-1/2 with better interference efficiency was chosen for further investigations. According to the results of CCK-8 assay, circZDBF2 knockdown substantially reduced the optical density (OD) value at 450 nm (Fig. 2A), implying that cell viability was repressed by silencing circZDBF2. Further, it was manifested from colony formation assay that cell proliferative capability was suppressed upon circZDBF2 depletion, as the number of cell colonies was obviously declined in the sh-circZDBF2-transfected groups (Fig. 2B). In addition, we performed transwell assay and found that the down-regulation of circZDBF2 caused a remarkable reduction in the number of invaded cells (Fig. 2C). Moreover, data of wound healing assay indicated that the relative wound width was increased in cells with circZDBF2 down-regulation (Fig. 2D), suggesting that cell migration was suppressed by circZDBF2 inhibition. Next, it was found that EMT phenotype was repressed by circZDBF2 knockdown (Fig. 2E). Western blot as well as qRT-PCR data further proved that circZDBF2 down-regulation elevated ZO-1 and E-cadherin expression while caused a decline on N-cadherin and Vimentin expression (Fig. 2F), demonstrating that EMT process was significantly hindered by circZDBF2 depletion in OSCC cells.

At the same time, we detected the effect of circZDBF2 up-regulation on OSCC cells after confirming the overexpression of circZDBF2 in SCC9 and SCC15 cells by transfecting pcDNA3.1-circZDBF2 (Additional file 1: Fig. S1C). Results of CCK-8 assay as well as colony formation assay manifested that circZDBF2 upregulation promoted cell viability and proliferation (Additional file 2: Fig. S2A, B). Further, cell invasion and migration was facilitated by up-regulating circZDBF2, as evidenced by transwell as well as wound healing assay (Additional file 2: Fig. S2C, D). Lastly, it was proved that EMT process was accelerated in the pcDNA3.1-circZDBF2 transfected cells (Additional file 2: Fig. S2E, F). Taken together, circZDBF2 accelerates the malignant cell behaviors in OSCC.

### CircZDBF2 acts as a sponge of miR-362-5p and miR-500b-5p in OSCC cells

**Fig. 3:**
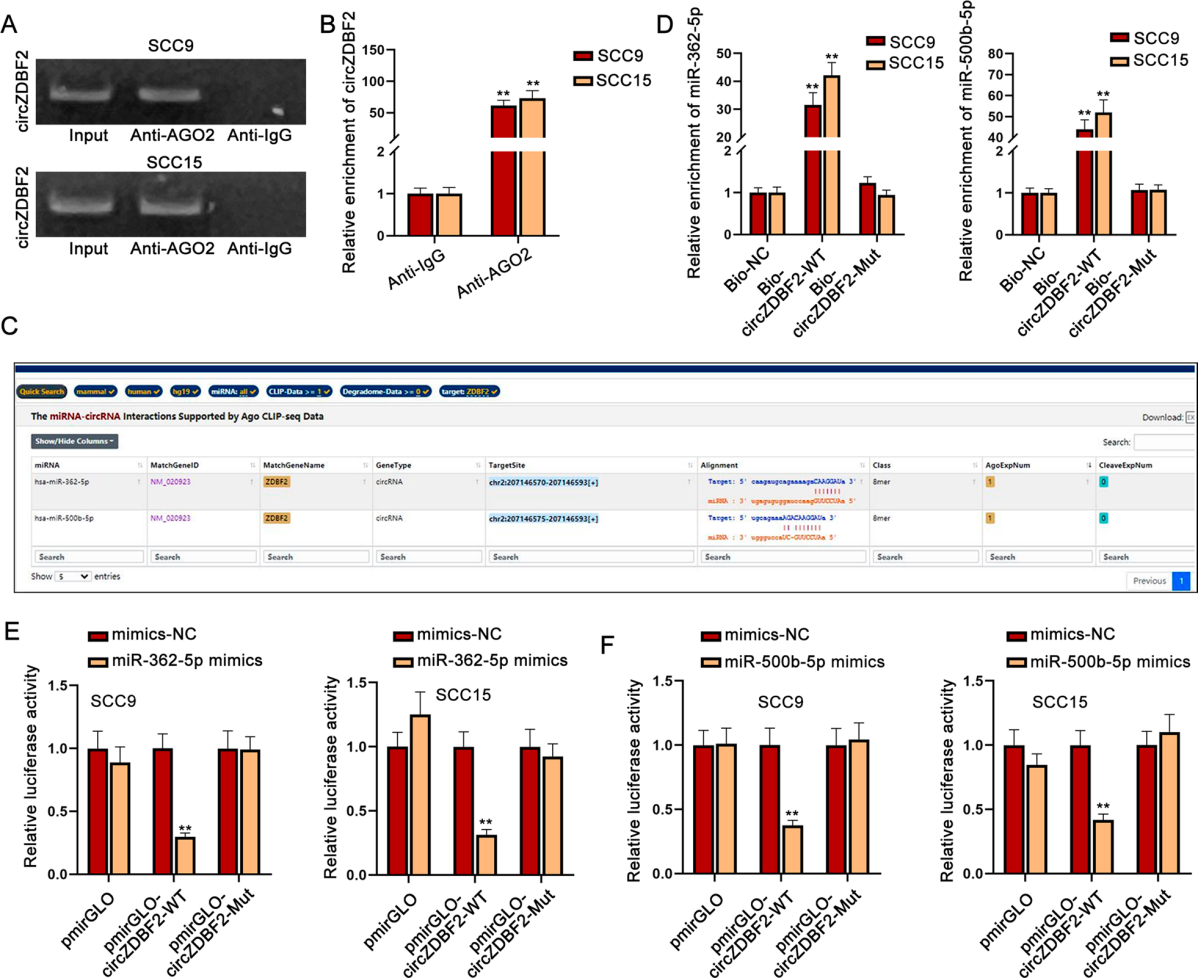
CircZDBF2 acts as a sponge of miR-362-5p and miR-500b-5p in OSCC cells. **A**, **B** The binding situation of circZDBF2 to AGO2 protein was evaluated by RIP assay. **C** ENCORI database was applied to predict the possible miRNAs for circZDBF2. **D** RNA pull down assay was taken to examine the enrichment of miR-362-5p or miR-500b-5p in Bio-circZDBF2 groups. **E**, **F** Luciferase reporter assay was applied to testify the combination of circZDBF2 with miR-362-5p or miR-500b-5p. ^**^P < 0.01

The competing endogenous RNA (ceRNA) network is a famous post-transcriptional regulatory mechanism of RNA interaction in cancers [20]. CircRNAs have been demonstrated to act as ceRNAs to sponge miRNAs and then modulate their downstream target genes, thereby accelerating or inhibiting cancer progression [21]. The result of AGO2-RIP assay manifested that circZDBF2 was enriched in anti-AGO2 group (Fig. 3A, B), suggesting that circZDBF2 can function as a ceRNA in OSCC. Next, we continued to discover the possible miRNAs of circZDBF2. Through ENCORI (http://starbase.sysu.edu.cn/index.php) database (low stringency), two miRNAs (miR-362-5p and miR-500b-5p) had the possibility to combine with circZDBF2 (Fig. 3C). As shown from RNA pull down assay, the biotinylated circZDBF2-WT probe abundantly pulled down miR-362-5p and miR-500b-5p (Fig. 3D). Moreover, the overexpression of miR-362-5p or miR-500b-5p in SCC9 and SCC15 cells reduced the luciferase activity of circZDBF2-WT but did not affect circZDBF2-Mut group (Fig. 3E, F). In short, circZDBF2 directly combined with miR-362-5p and miR-500b-5p in OSCC cells.

### RNF145 is the target gene of miR-362-5p and miR-500b-5p

**Fig. 4:**
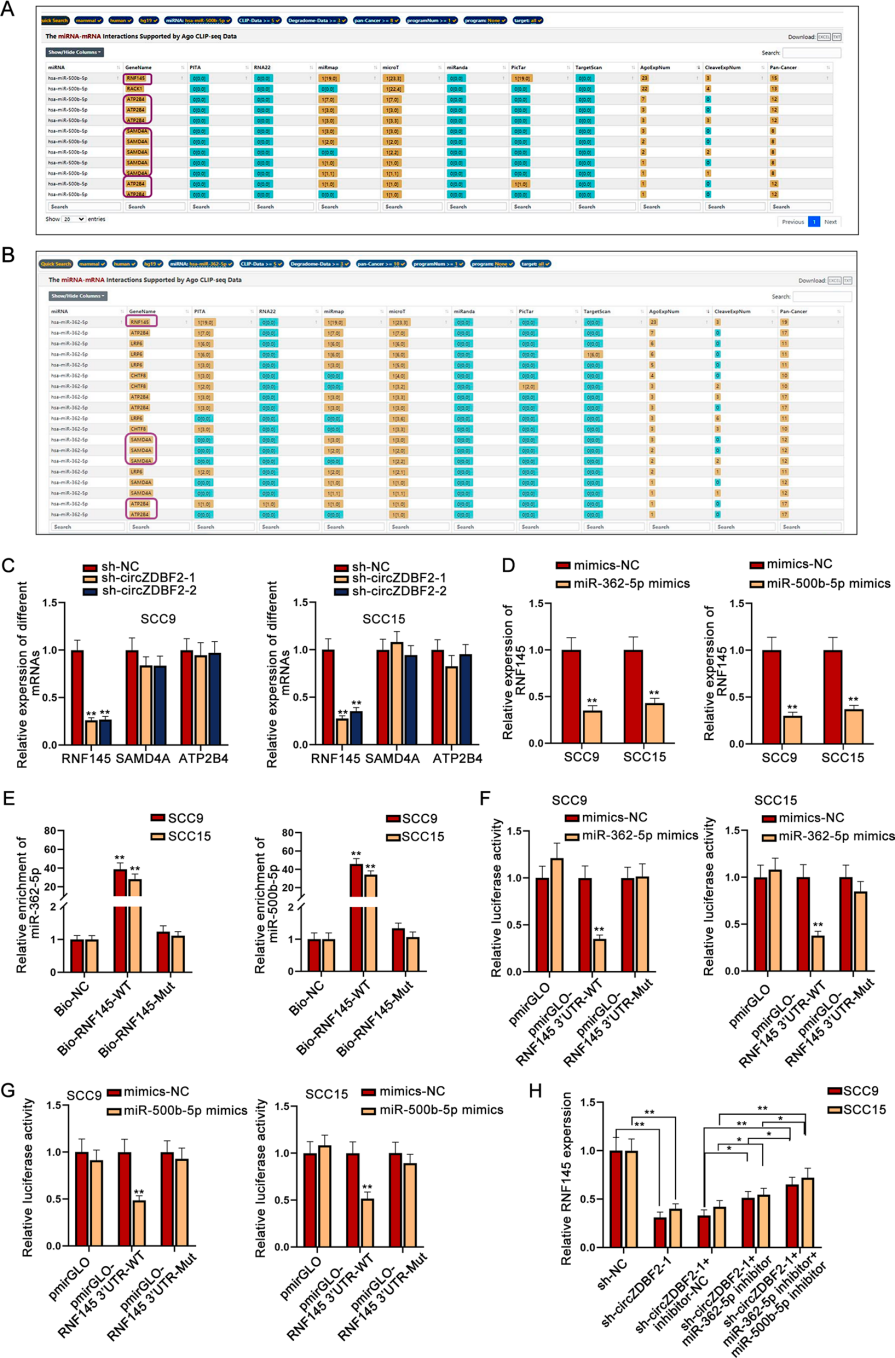
RNF145 is the target gene of miR-362-5p and miR-500b-5p. **A**, **B** Possible mRNAs of miR-362-5p and miR-500b-5p were forecast through ENCORI database. **C** RNF145, SAMD4A and ATP2B4 expression was detected by qRT-PCR after circZDBF2 was knocked down in OSCC cells. **D** The expression of RNF145 was detected by qRT-PCR in OSCC cells transfected with miR-362-5p mimics or miR-500b-5p mimics. **E**–**G** RNA pull down assay, together with luciferase reporter assay was performed to prove the binding of RNF145 with miR-362-5p or miR-500b-5p. **H** RNF145 expression was detected via qRT-PCR in different groups transfected with sh-NC, sh-circZDBF2-1, sh-circZDBF2-1 + inhibitor-NC, sh-circZDBF2-1 + miR-362-5p inhibitor and sh-circZDBF2-1 + miR-362-5p inhibitor + miR-500b-5p inhibitor plasmids. ^*^P < 0.05, ^**^P < 0.01

Through ENCORI database with the specific condition (strict stringency; 10 cancer types), three possible mRNAs were observed to be shared by miR-362-5p and miR-500b-5p, which were RNF145, SAMD4A (sterile alpha motif domain containing 4A) and ATP2B4 (ATPase plasma membrane Ca2+ transporting 4) (Fig. 4A, B). Among them, only RNF145 was declined by circZDBF2 silencing, while the expression of the other two mRNAs was not affected (Fig. 4C). Therefore, we selected RNF145 for later experiments. qRT-PCR data further proved that RNF145 expression was declined by miR-362-5p overexpression or miR-500b-5p overexpression in SCC9 and SCC15 cells (Fig. 4D), which supported the ceRNA mode. Next, the binding capacity between RNF145 and miR-362-5p/miR-500b-5p was evidenced by RNA pull down result (Fig. 4E). Subsequently, it was discovered that the up-regulation of miR-362-5p or miR-500b-5p reduced the luciferase activity of RNF145 3’UTR-WT rather than the mutant group (Fig. 4F, G). It was shown that circZDBF2 knockdown did not affect miR-362-5p or miR-500b-5p expression (Additional file 3: Fig. S3A). Further, RNF145 expression inhibited by circZDBF2 knockdown was partially rescued by the co-inhibition of miR-362-5p and miR-500b-5p in OSCC cells (Fig. 4H), suggesting that circZDBF2 might have another pathway to regulate RNF145 level. Overall, RNF145 is the target gene of miR-362-5p and miR-500b-5p in OSCC cells.

### CircZDBF2 contributes to RNF145 transcription by recruiting CEBPB

**Fig. 5:**
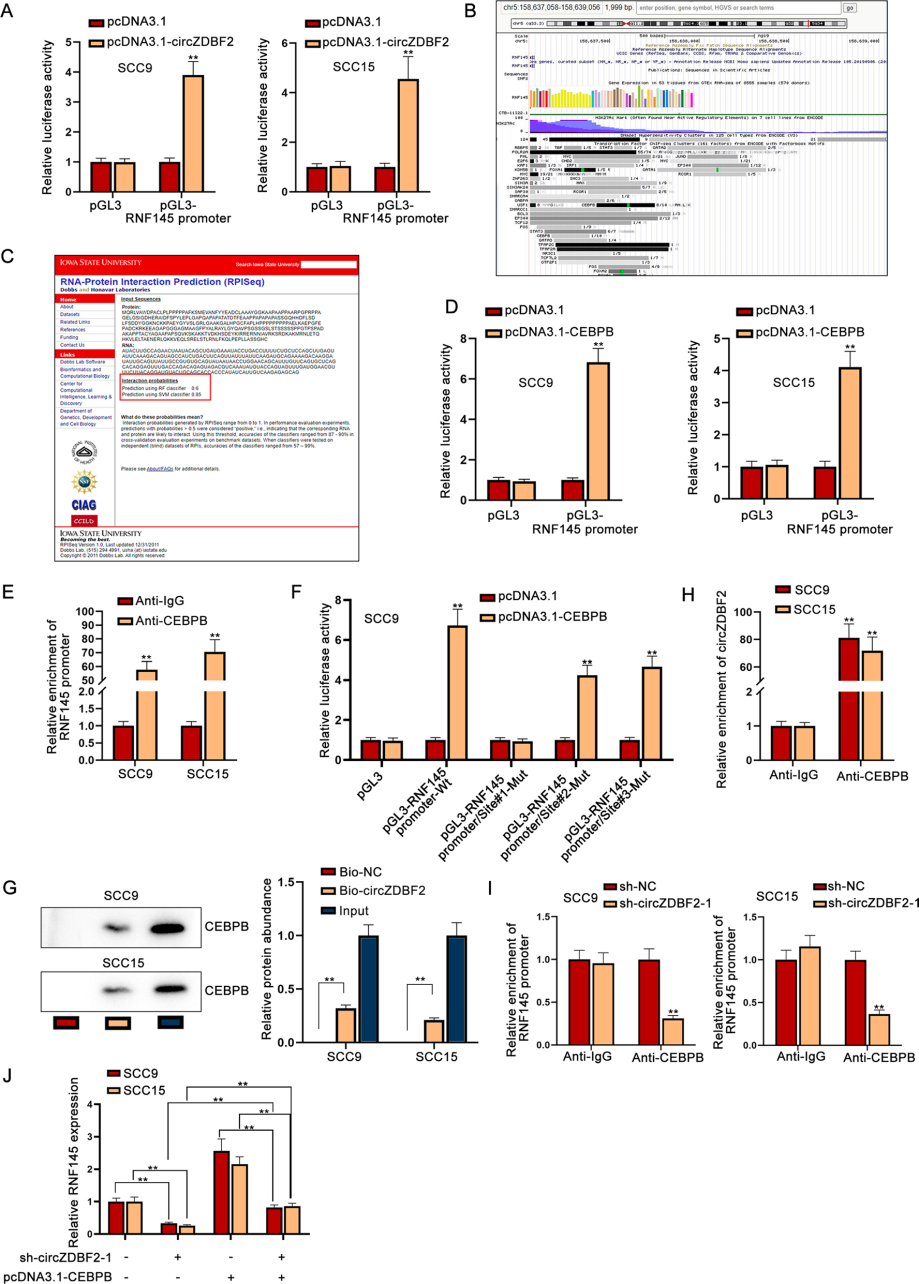
CircZDBF2 regulates RNF145 by recruiting CEBPB. **A** Luciferase reporter assay was taken to analyze the binding situation of circZDBF2 to RNF145 promoter. **B** UCSC database was applied to predict the possible transcription factors for circZDBF2. **C** The possibility of circZDBF2 combining with CEBPB was predicted through RPISeq database. **D** Luciferase reporter assay was applied to detect the binding of CEBPB to RNF145 promoter. **E** The enrichment of RNF145 promoter in anti-IgG group and anti-CEBPB group was measured by ChIP assay. **F** Luciferase reporter assay was taken to verify the specific sites that CEBPB bound to RNF145 promoter. **G** The enrichment of CEBPB with or without the biotinylated circZDBF2 probe was measured by RNA pull down assay. **H** CircZDBF2 enrichment in anti-IgG group and anti-CEBPB group was analyzed by RIP assay. **I** The relative enrichment of RNF145 promoter in anti-IgG group and anti-CEBPB group was measured by ChIP assay upon circZDBF2 downregulation. **J** RNF145 expression was detected by qRT-PCR in different groups. ^**^P < 0.01

Since miR-362-5p and miR-500b-5p could not completely rescue the regulatory effect of circZDBF2 on RNF145, we speculated that circZDBF2 may have other pathways to regulate RNF145. We had verified that circZDBF2 was also distributed in the nucleus of OSCC cells, and it was manifested through dual luciferase reporter gene experiments that circZDBF2 up-regulation elevated the luciferase activity of RNF145 promoter (Fig. 5A), so we suspected that circZDBF2 in the nucleus might regulate RNF145 transcription. We utilized UCSC (http://genome.ucsc.edu/) website to predict the possible transcription factors for RNF145 and found that MYC (MYC proto-oncogene), E2F6 (E2F transcription factor 6), FOXA1 (forkhead box A1), FOXA2 (forkhead box A2), FOS (proto-oncogene c-Fos), ZNF263 (zinc finger protein 263), GABPA (GA binding protein transcription factor subunit alpha), USF1 (upstream transcription factor 1), TCF12 (transcription factor 12), TFAP2A (transcription factor AP-2 alpha) and CEBPB may bind to RNF145 (Fig. 5B). In accordance with the existing studies, we discovered that MYC [22], E2F6 [23], FOXA1 [24] and CEBPB [25] expressed at a high level in OSCC cell lines. Therefore, we selected these four transcription factors for further screening. Through qRT-PCR, RNF145 expression was only declined by CEBPB down-regulation rather than the other candidates (Additional file 4: Fig. S4A). Further, RNA–Protein Interaction Prediction (RPISeq; http://pridb.gdcb.iastate.edu/RPISeq/) was applied to predict the potential interaction between circZDBF2 and CEBPB (Fig. 5C). Based on these results, we speculated that circZDBF2 affected RNF145 transcription through interacting with CEBPB. We up-regulated CEBPB expression via transfecting the pcDNA3.1-CEBPB plasmids into OSCC cells (Additional file 1: Fig. S1D), and it was uncovered that CEBPB overexpression significantly elevated the luciferase activity of RNF145 promoter (Fig. 5D). Data of ChIP assay further showed that RNF145 promoter was enriched by anti-CEBPB (Fig. 5E), which further proved the binding of CEBPB to RNF145 promoter. Next, JASPAR (http://jaspar.genereg.net/) database was utilized to predict the binding sites of CEBPB to RNF145 promoter, and three reliable CEBPB-binding sites (Site#1/2/3) were exhibited (Additional file 4: Fig. S4B). Further, it was revealed that CEBPB overexpression increased the luciferase activity of RNF145 promoter-WT, RNF145 promoter/Site#2-Mut and RNF145 promoter/Site#3-Mut while RNF145 promoter/Site#1-Mut group was not affected (Fig. 5F), indicating that CEBPB bound to RNF145 promoter at Site 1. Next, the interactivity between CEBPB and circZDBF2 was certified through RNA pull down and RIP assays (Fig. 5G, H). Interestingly, we disclosed that after the knockdown of circZDBF2, the enrichment of RNF145 promoter precipitated by CEBPB antibody decreased significantly (Fig. 5I). Furthermore, it was shown that RNF145 expression was repressed by circZDBF2 depletion or increased by CEBPB overexpression, and the decreased RNF145 expression caused by circZDBF2 knockdown was countervailed by the addition of CEBPB (Fig. 5J). In short, circZDBF2 enhances RNF145 transcription by recruiting CEBPB.

### RNF145 accelerates OSCC cell proliferation, migration, invasion and EMT process

It was shown from qRT-PCR assay that RNF145 expressed at a higher level in OSCC cells than in the normal HOK cell line (Additional file 5: Fig. S5A). We separately inhibited RNF145 expression via sh-RNF145-1/2/3 transfection or elevated RNF145 expression via pcDNA3.1-RNF145 transfection before conducting loss- and gain-of-function experiments (Additional file 1: Fig. S1E, F). Through CCK-8 along with colony formation assay, it was uncovered that the down-regulation of RNF145 obviously repressed cell proliferation, while the overexpression of RNF145 promoted cell proliferation (Additional file 5: Fig. S5B, C and Additional file 6: Fig. S6A, B). Subsequently, cell invasion and migration was suppressed by silencing RNF145, while accelerated by overexpressing RNF145 (Additional file 5: Fig. S5D, E and Additional file 6: Fig. S6C, D). Likewise, we discovered that the knockdown of RNF145 exerted an inhibitory effect on EMT phenotype, while up-regulation of RNF145 facilitated EMT process (Additional file 5: Fig. S5F, G and Additional file 6: Fig. S6E, F). Altogether, RNF145 exerts a tumor-facilitating function in OSCC.

### CircZDBF2 facilitates the progression of OSCC via up-regulating RNF145

**Fig. 6:**
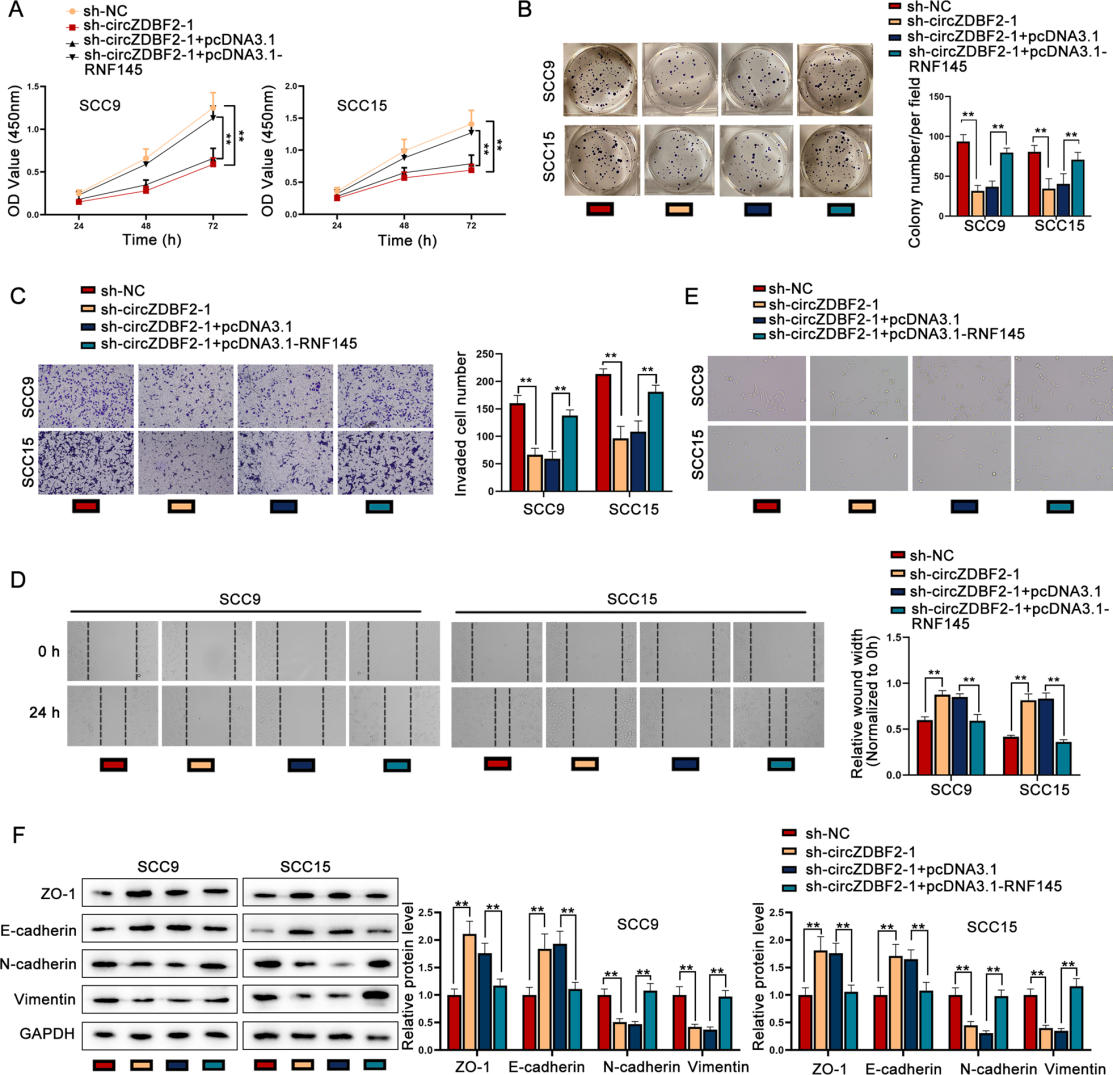
CircZDBF2 facilitates the progression of OSCC via up-regulating RNF145. **A**, **B** OSCC cell proliferative capability was detected by CCK-8 assay as well as colony formation assay in different groups transfected with sh-NC, sh-circZDBF2-1, sh-circZDBF2-1 + pcDNA3.1 and sh-circZDBF2-1 + pcDNA3.1-RNF145. **C**, **D** Transwell assay, together with wound healing assay was applied to evaluate cell invasion and migration under different transfection conditions. **E**, **F** EMT phenotype was detected in different transfection groups. ^**^P < 0.01

Finally, the regulatory effect of circZDBF2/RNF145 axis on OSCC progression was testified through rescue functional assays. We observed that the proliferation of OSCC cells was suppressed by the transfection of sh-circZDBF2-1, while such effect was totally recovered by the co-transfection of sh-circZDBF-1 and pcDNA3.1-RNF145 (Fig. 6A, B). Next, results of transwell assay as well as wound healing assay manifested that cell invasion and migration inhibited by circZDBF2 knockdown was fully rescued by RNF145 up-regulation (Fig. 6C, D). Moreover, RNF145 overexpression counteracted the inhibitory effect of silencing circZDBF2 on EMT process (Fig. 6E, F). In a word, circZDBF2 facilitates the progression of OSCC via up-regulating RNF145.

### RNF145 activates NFκB pathway to positively modulate IL-8

It has been reported that ring finger protein 183 (RNF183) can induce the activation of protein p65 in the NFκB signaling pathway to regulate the transcription of IL-8 [26]. IL-8 has been shown to promote the tumorigenesis of OSCC [27, 28]. RNF145 and RNF183 are homologous proteins, so we speculated that RNF145 can also induce p65 so as to activate the NFκB signaling pathway and regulate the transcription of IL-8. Through western blot along with qRT-PCR, we found that the down-regulation of RNF145 repressed the protein level of p65 while increased that of IκBɑ. By contrast, the protein level of p65 was promoted while that of IκBɑ was inhibited by the up-regulation of RNF145 (Additional file 7: Fig. S7A, B). It was then shown by qRT-PCR that IL-8 expression was declined in sh-RNF145-transfected OSCC cells while increased in pcDNA3.1-RNF145-trnasfected cells (Additional file 7: Fig. S7C, D). Further, it was demonstrated from luciferase reporter assays that the luciferase activity of IL-8 promoter was elevated by the upregulation of wild-type RNF145 rather than mutant RNF145 (Additional file 7: Fig. S7E), suggesting that RNF145 promoted the transcription of IL-8 in OSCC. In a word, RNF145 activates NFκB pathway to transcriptionally facilitate IL-8 expression.

### CircZDBF2 promotes tumor growth of OSCC in vivo

**Fig. 7:**
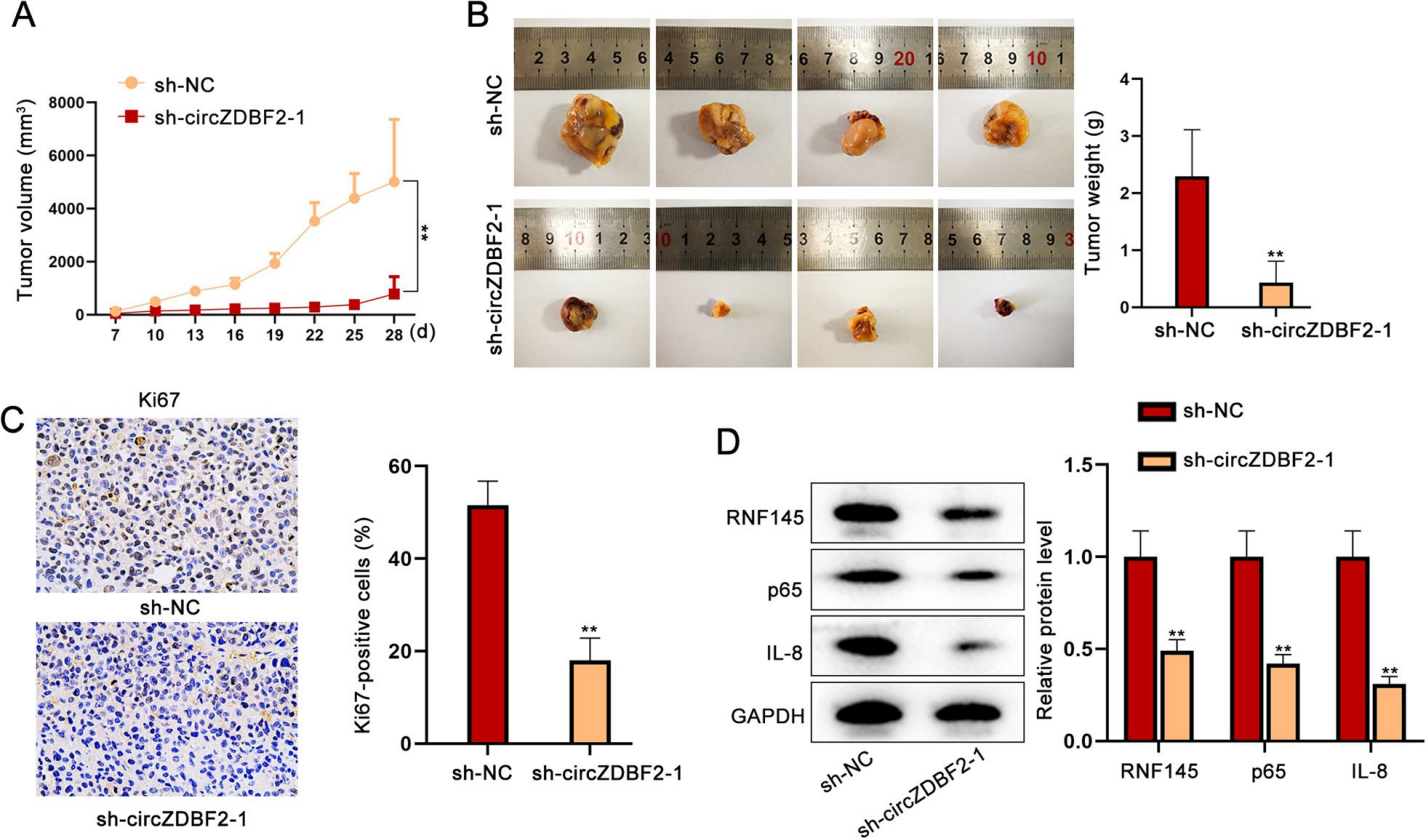
CircZDBF2 promotes tumor growth of OSCC in vivo*.* **A**, **B** The effects of circZDBF2 silencing on in vivo tumor volume and weight were analyzed. **C** Ki67 expression in tumor tissues was detected by immunohistochemistry assay after circZDBF2 was silenced. **D** The expression level of RNF145, p65 and IL-8 was detected in negative control group and sh-circZDBF2-1 group. ^**^P < 0.01

We detected the influence circZDBF2 may exert on OSCC tumor growth by xenotransplantation assay. Results manifested that tumor growth tendency was slower in the group with circZDBF2-silenced OSCC cells than in the negative control group (Fig. 7A). Besides, the tumor size along with tumor weight was decreased in the sh-circZDBF2 transfection groups (Fig. 7B). The expression of Ki67 (the nuclear antigen representing cell proliferation) was detected by immunohistochemistry, which showed that the positivity of Ki67 was decreased by circZDBF2 downregulation (Fig. 7C). Furthermore, the expression of RNF145, p65 and IL-8 was decreased in tumors with inhibited circZDBF2 expression (Fig. 7D). In conclusion, circZDBF2 accelerates in vivo OSCC tumor growth by regulating RNF145, p65 and IL-8.

## Discussion

OSCC is a common squamous cell carcinoma of the head and neck with high incidence. In the last decades, molecular markers have been accepted to be important for the diagnosis, prognosis and treatment of cancers, including proteins [29–31] and miRNAs [32]. In recent years, circRNAs gradually become molecular markers for many cancers, as a plenty of circRNA have also been proven to play crucial roles during the development of cancers including OSCC. For example, circ-PKD2 targets miR-204-3p and APC2 (APC regulator of WNT signaling pathway 2) to suppress the carcinogenesis of OSCC [33]. Circ-ABCB10 accelerates the malignant progression of OSCC by absorbing miR-145-5p [34]. In our research, the specific role of circZDBF2 was explored in OSCC. CircZDBF2 was a novel circRNA and it was shown through GEO database that it was down-regulated in OSCC tissues. CircZDBF2 depletion was verified to impair cell proliferation, invasion, migration as well as EMT process in OSCC. The carcinogenic property of circZDBF2 is identified for the first time.

Recently, ceRNA regulatory mechanism in human cancers has arisen more and more attention. The ceRNA network describes the interaction among different RNA species, including circRNAs [35]. It refers to that circRNAs can act as ceRNAs to sponge miRNAs, thereby releasing the inhibition of miRNAs on mRNAs expression at post-transcriptional level [35, 36]. They communicate with and co-regulate each other by competing for binding to shared miRNAs [36]. This circRNA-miRNA-mRNA regulatory axis has been identified in a variety of human cancer cells [20]. For instance, circ_0001421 promotes glycolysis and lung cancer development by regulating miR-4677-3p to upregulating cell division cycle associated 3 (CDCA3) [37]. Circ_0084927 sponges miR-142-3p and up-regulates ADP ribosylation factor like GTPase 2 (ARL2) to aggravate the proliferation of cervical cancer cells [38]. As a mechanism of post-transcriptional regulation, the prerequisite of ceRNA is that circRNA mainly located in cytoplasm. Through the results of FISH assay, the cytoplasm location of circZDBF2 in OSCC cells was determined. After that, ENCORI (The Encyclopedia of RNA Interactomes) database was applied to predict the possible miRNAs for circZDBF2 and performed a serious of mechanism assays to verify the combination between genes. Ultimately, we proved that circZDBF2 could function as a ceRNA to sponge miR-362-5p and miR-500b-5p in OSCC cells. Previously, miR-362-5p was reported to represses neuroblastoma malignancy by targeting phosphatidylinositol-4-phosphate 3-kinase catalytic subunit type 2 beta (PI3K-C2β) [39]. MiR-362-5p also enhances the cisplatin sensitivity of gastric cancer cells via SUZ12 polycomb repressive complex 2 subunit (SUZ12) [40]. Importantly, it is reported that miR-362-5p exerts a repressive impact on OSCC cell proliferation, migration as well as invasion, which is consistent with our findings. In addition, miR-500b-5p has been illustrated to participate in the occurrence and development of diseases. For example, circMACF1 has an inhibitory effect on acute myocardial infarction via miR-500b-5p and EMP1 (epithelial membrane protein 1) [41]. However, little research has been made on the regulatory role of miR-500b-5p in human cancers. It is verified for the first time that miR-500b-5p mediated OSCC development by acting as a cancer inhibitor.

RNF145 is an E3 ubiquitin ligase, and its homologous family RNF183 can induce the activation of NFκB signaling pathway to regulate the transcription of IL-8 [26]. In the current study, through mechanism assays, we proved that miR-362-5p and miR-500b-5p could directly target RNF145 in OSCC cells. High expression of RNF145 was verified in OSCC cells and it was proven that RNF145 downregulation inhibited OSCC malignancy. Further, qRT-PCR data manifested that RNF145 was positively correlated with circZDBF2 while negatively correlated with miR-362-5p and miR-500b-5p. The experimental results of rescue assay testified that the inhibition of miR-362-5p and miR-500b-5p did not totally rescue the effect of circZDBF2 knockdown on RNF145 expression, suggesting that there existed another pathway for circZDBF2 to regulate RNF145 expression. Previous FISH assays have proved that circZDBF2 is also distributed in the nucleus of OSCC cells, so we speculated that circZDBF2 may regulate RNF145 by recruiting certain transcription factors. Through bioinformatics prediction tools along with mechanism experiments, CEBPB was selected and verified to be the target transcription factor of RNF145 in OSCC cells. Previous studies have indicated that CEBPB expressing at a high level in OSCC cell lines [25] can be taken as the transcription factor regulating downstream genes [42]. In short, it was verified that circZDBF2 up-regulated RNF145 expression by recruiting the transcription factor CEBPB in OSCC.

NFκB signaling pathway is increasingly recognized as a crucial modulator during cancer initiation and progression [43]. NFκB modulates gene expression and different cell behaviors of cancers, which include proliferation, migration and invasion [44]. Aberrant NFκB signaling has shown to be associated with different types of cancers, including lung cancer, prostate cancer, and ovarian cancer [45]. For example, miR-210-3p promotes the EMT process as well as metastasis of prostate cancer cells via NFκB signaling [46]. P50 and p65 are the important members of NFκB family. The primary mechanism of canonical NFκB activation is the degradation of IκBα [47]. It is reported that RNF183 can induce the activation of protein p65 in the NFκB signaling pathway to regulate the transcription of IL-8 [26]. IL-8 has been shown to promote the tumorigenesis of OSCC [27, 28]. RNF145 and RNF183 are homologous proteins, so we speculated that RNF145 can also activate the NFκB signaling pathway through the same way. Through western blot assay, we found that down-regulation of RNF145 repressed the protein level of p50 and p65 while increased that of IκBɑ. It was further proven that RNF145 promoted the transcription of IL-8. Overall, these findings confirmed that RNF145 regulated OSCC progression via activating NFκB signaling pathway and regulating the transcription of IL-8. IL-8 has been identified as one of the target genes playing important roles in NFκB pathway [48], and our findings may help to provide more strategies for the IL-8-NFκB pathway exploration in the future.

## Conclusion

It is verified for the first time that up-regulated circZDBF2 promotes the malignant cell behaviors in OSCC including cell proliferation, invasion, migration as well as the EMT process. Moreover, circZDBF2 functioning as a ceRNA to sponge miR-362-5p and miR-500b-5p to up-regulate RNF145 level in OSCC cells is revealed. Further, circZDBF2 can also recruit the transcription factor CEBPB to up-regulate RNF145 expression, and RNF145 is verified to activate NFκB signaling pathway and regulate IL-8 transcription in OSCC. The main findings of our study have been summarized in a graphical abstract. For the limitations of our research, it is worth to further investigate the upstream mechanism of circZDBF2 and repeat the experiments in other OSCC cell lines; also, the collection of clinical samples will certainly help to complete our study in the future. In summary, we hope that these findings may help to create new OSCC therapy.

## Supplementary Information

**Additional file 1: Figure S1.** (A) GSE145608 revealed the expression profile of hsa_circRNA_002141 (circZDBF2) in oral cancer cell lines and human normal control cell line. (B) CircZDBF2 expression was silenced in SCC9 and SCC15 cells via the transfection of sh-circZDBF2-1/2/3. (C) CircZDBF2 expression was enhanced by the transfection of pcDNA3.1-circZDBF2 in SCC9 and SCC15 cells. (D) CEBPB expression was elevated in SCC9 and SCC15 cells by the pcDNA3.1-CEBPB transfection. (E) RNF145 expression was silenced in SCC9 and SCC15 cells by the transfection of sh-RNF145-1/2/3. (F) RNF145 expression was enhanced in SCC9 and SCC15 cells via the pcDNA3.1-RNF145 transfection. ^**^P < 0.01.

**Additional file 2: Figure S2.** (A-B) CCK-8 assay as well as colony formation assay was employed to estimate cell viability and proliferation when circZDBF2 was overexpressed. (C-D) Transwell assay, together with wound healing assay was performed to measure cell invasion and migration upon circZDBF2 upregulation. (E–F) EMT phenotype in SCC9 and SCC15 cells transfected with pcDNA3.1-circZDBF2 was detected. ^*^P < 0.05, ^**^P < 0.01.

**Additional file 3: Figure S3.** (A) The expression of miR-362-5p or miR-500b-5p was detected by qRT-PCR in OSCC cells transfected with sh-circZDBF2-1/2.

**Additional file 4: Figure S4.** (A) RNF145 expression was detected by qRT-PCR in different groups. (B) JASPAR database was applied to predict the binding sites of RNF145 to CEBPB. ^**^P < 0.01.

**Additional file 5: Figure S5.** (A) RNF145 expression in OSCC cell lines (SCC9, SCC15, SCC25) and HOK cell line was tested by qRT-PCR analysis. (B-C) CCK-8 assay as well as colony formation assay was carried out to evaluate cell proliferation upon RNF145 upregualtion. (D-E) Transwell assay, together with wound healing assay was taken to estimate the influence of RNF145 upregulation on OSCC cell invasion and migration. (F-G) EMT phenotype in SCC9 and SCC15 cells transfected with pcDNA3.1-RNF145 was assessed. ^**^P < 0.01.

**Additional file 6: Figure S6.** (A, B) CCK-8 and colony formation assays were applied for measuring cell viability and proliferation when RNF145 was silenced. (C, D) Transwell assay and wound healing assay were utilized to assess cell invasion and migration when RNF145 was inhibited in OSCC cells. (E, F) EMT phenotype was detected in sh-RNF145-1/2-transfected SCC9 and SCC15 cells. ^**^P < 0.01.

**Additional file 7: Figure S7.** (A, B) Western blot as well as qRT-PCR was used to detect the expression level of P50, P65 and IκBɑ after RNF145 was silenced or overexpressed in OSCC cells. (C, D) IL-8 expression was detected in OSCC cells after RNF145 was silenced or overexpressed. (E) Luciferase reporter assay was taken to analyze the regulation of IL-8 transcription before and after RNF145 knockdown in OSCC cells. ^*^P < 0.05, ^**^P < 0.01.

**Additional file 8: Table S1.** Primer sequences for indicated genes tested by qRT-PCR.

## Data Availability

Not applicable.
