# Supplementary figures and images for: CircZDBF2 up-regulates RNF145 by ceRNA model and recruits CEBPB to accelerate oral squamous cell carcinoma progression via NFκB signaling pathway
Source: J Transl Med. 2022 Apr 1;20:148. doi: 10.1186/s12967-022-03347-1 (PMC8973790; doi:10.1186/s12967-022-03347-1)

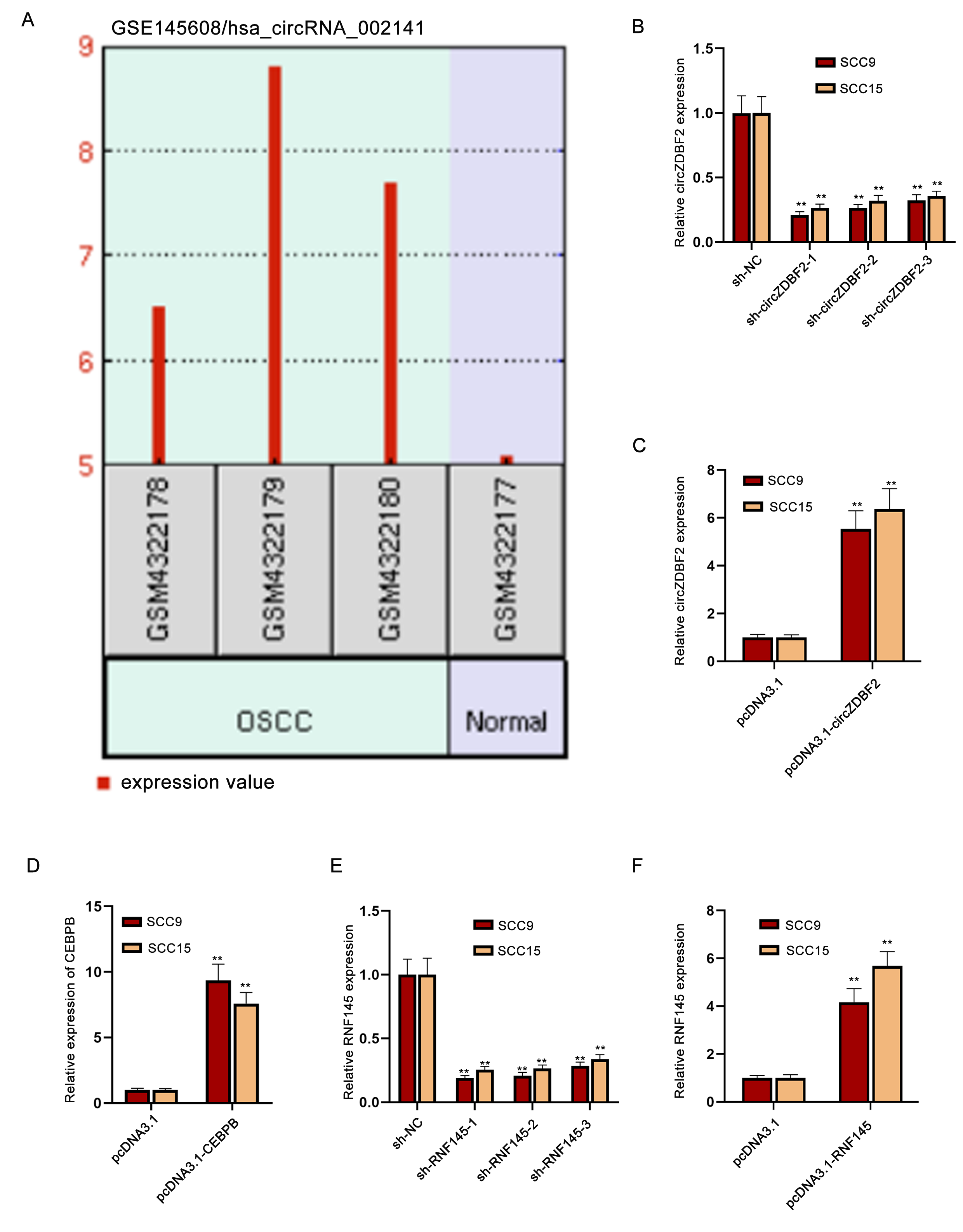

Supplement: Supplementary file 1 — Additional file 1: Figure S1. (A) GSE145608 revealed the expression profile of hsa_circRNA_002141 (circZDBF2) in oral cancer cell lines and human normal control cell line. (B) CircZDBF2 expression was silenced in SCC9 and SCC15 cells via the transfection of sh-circZDBF2-1/2/3. (C) CircZDBF2 expression was enhanced by the transfection of pcDNA3.1-circZDBF2 in SCC9 and SCC15 cells. (D) CEBPB expression was elevated in SCC9 and SCC15 cells by the pcDNA3.1-CEBPB transfection. (E) RNF145 expression was silenced in SCC9 and SCC15 cells by the transfection of sh-RNF145-1/2/3. (F) RNF145 expression was enhanced in SCC9 and SCC15 cells via the pcDNA3.1-RNF145 transfection. **P < 0.01. [file 12967_2022_3347_MOESM1_ESM.tif]

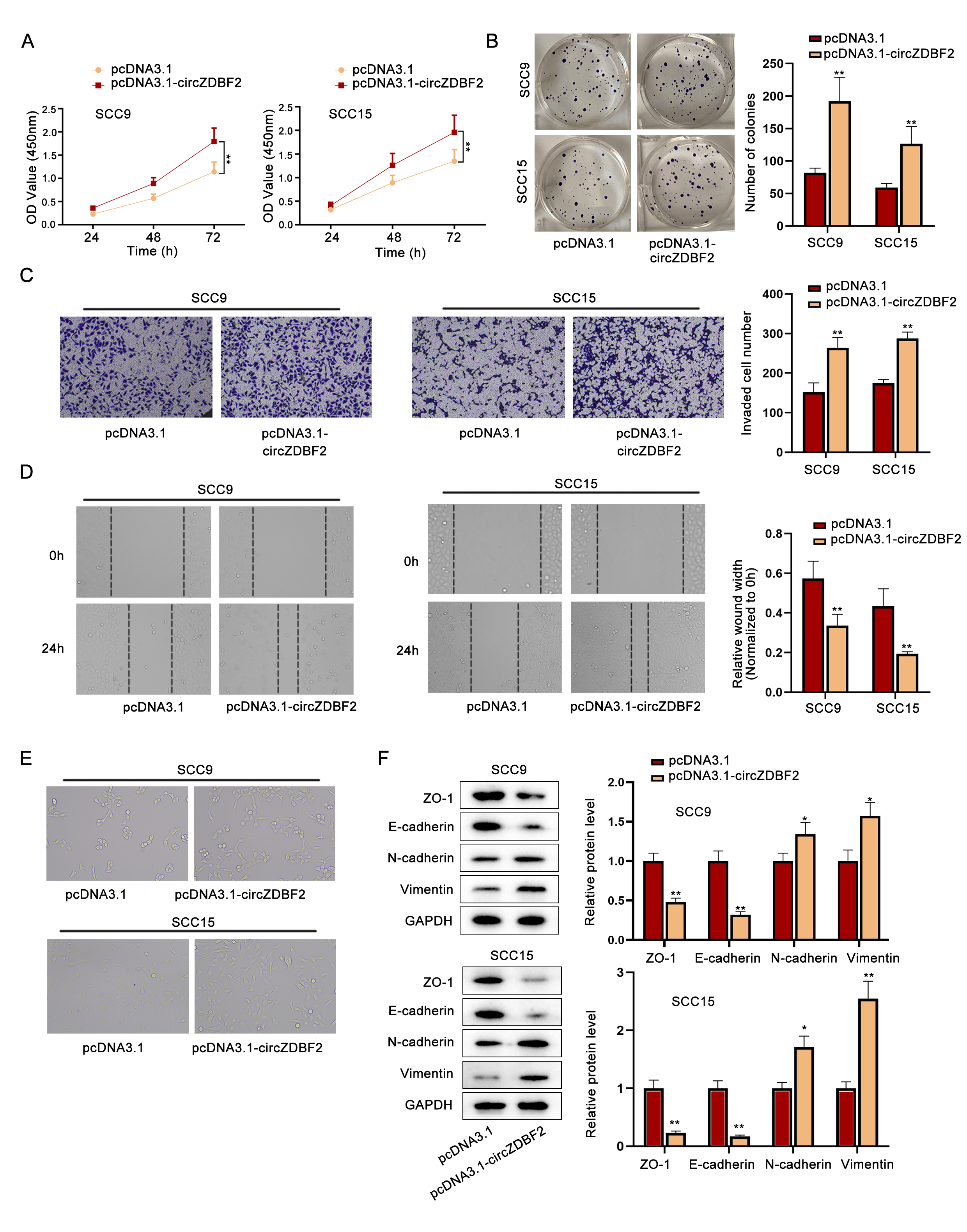

Supplement: Supplementary file 2 — Additional file 2: Figure S2. (A-B) CCK-8 assay as well as colony formation assay was employed to estimate cell viability and proliferation when circZDBF2 was overexpressed. (C-D) Transwell assay, together with wound healing assay was performed to measure cell invasion and migration upon circZDBF2 upregulation. (E–F) EMT phenotype in SCC9 and SCC15 cells transfected with pcDNA3.1-circZDBF2 was detected. *P < 0.05, **P < 0.01. [file 12967_2022_3347_MOESM2_ESM.tif]

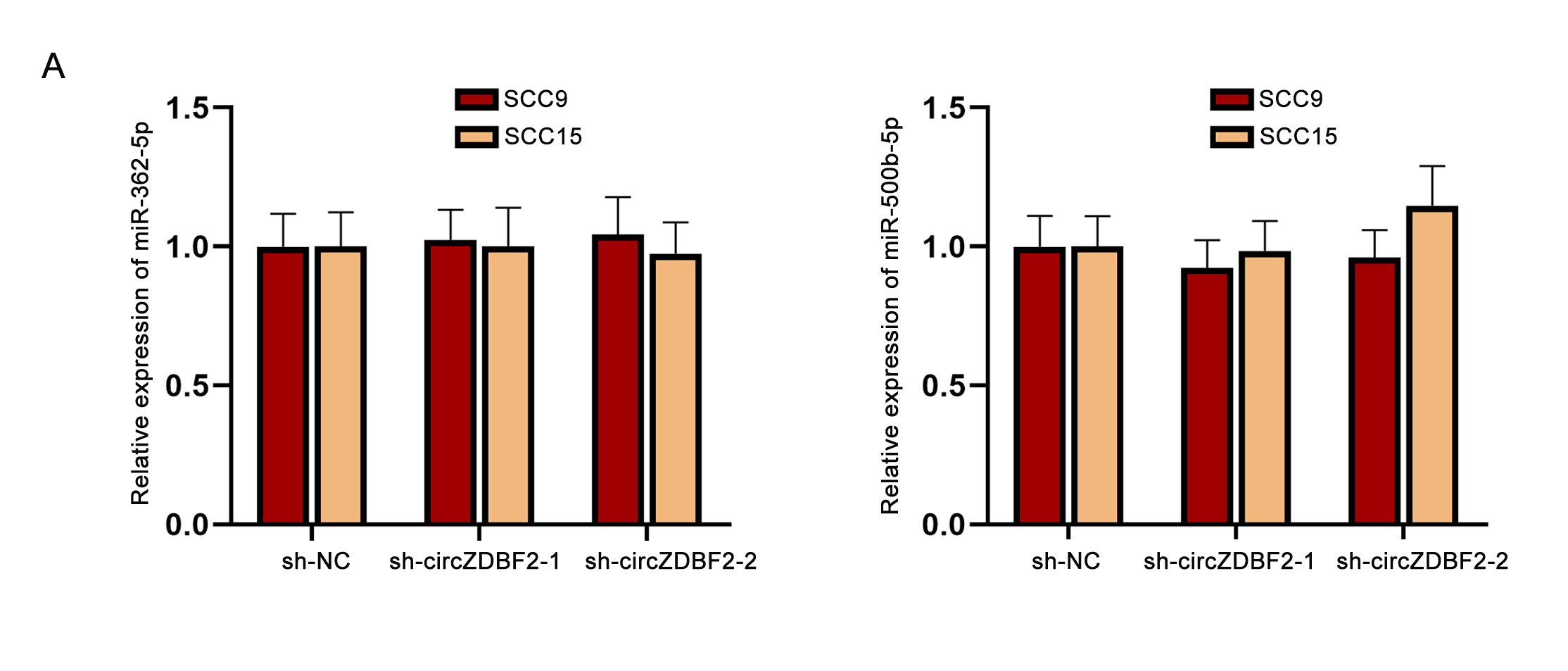

Supplement: Supplementary file 3 — Additional file 3: Figure S3. (A) The expression of miR-362-5p or miR-500b-5p was detected by qRT-PCR in OSCC cells transfected with sh-circZDBF2-1/2. [file 12967_2022_3347_MOESM3_ESM.tif]

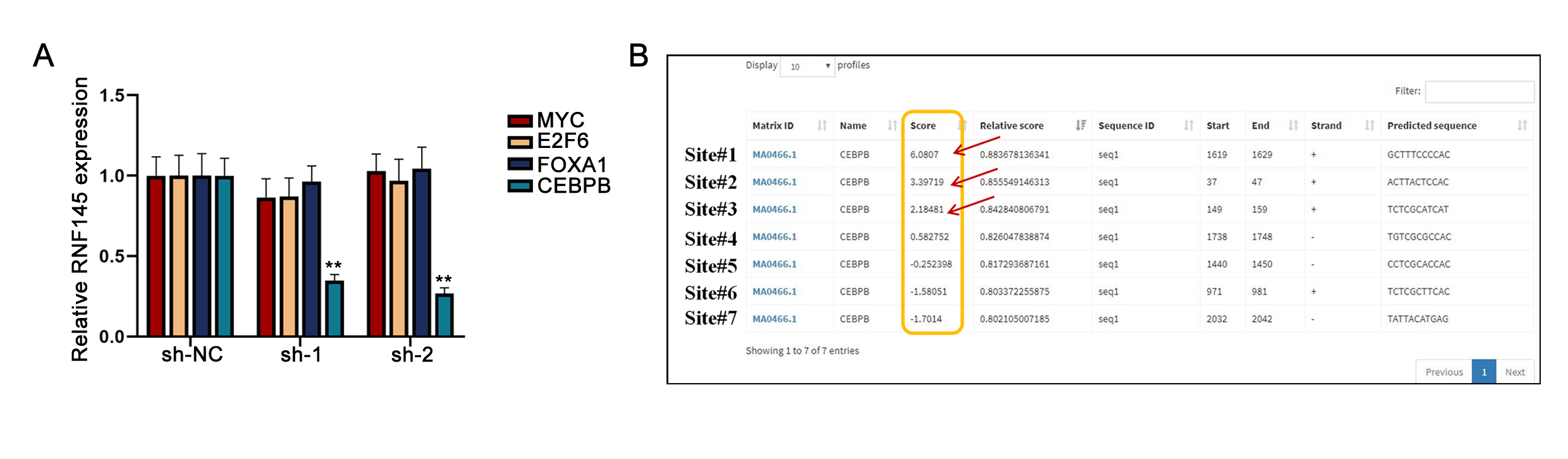

Supplement: Supplementary file 4 — Additional file 4: Figure S4. (A) RNF145 expression was detected by qRT-PCR in different groups. (B) JASPAR database was applied to predict the binding sites of RNF145 to CEBPB. **P < 0.01. [file 12967_2022_3347_MOESM4_ESM.tif]

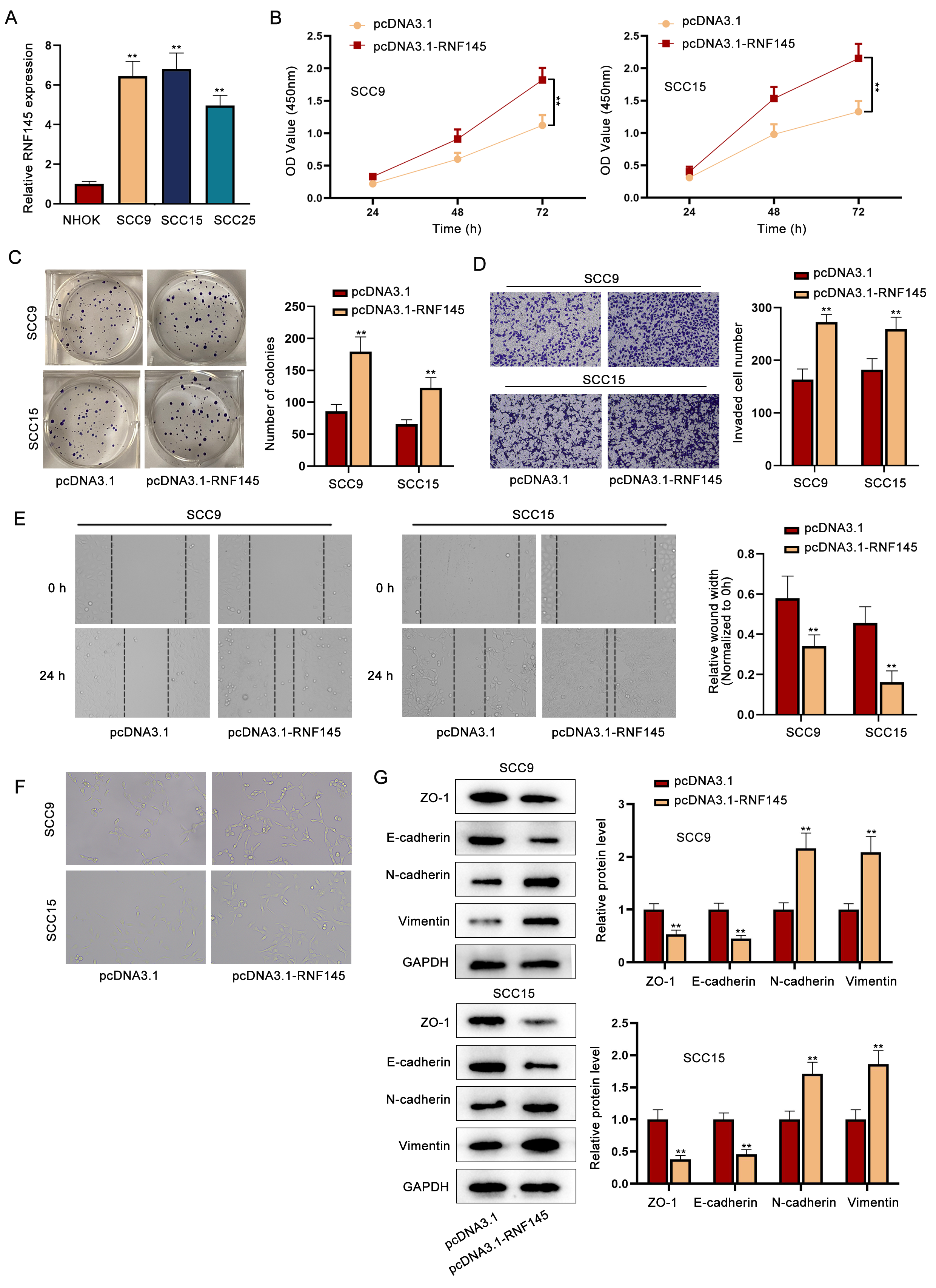

Supplement: Supplementary file 5 — Additional file 5: Figure S5. (A) RNF145 expression in OSCC cell lines (SCC9, SCC15, SCC25) and HOK cell line was tested by qRT-PCR analysis. (B-C) CCK-8 assay as well as colony formation assay was carried out to evaluate cell proliferation upon RNF145 upregualtion. (D-E) Transwell assay, together with wound healing assay was taken to estimate the influence of RNF145 upregulation on OSCC cell invasion and migration. (F-G) EMT phenotype in SCC9 and SCC15 cells transfected with pcDNA3.1-RNF145 was assessed. **P < 0.01. [file 12967_2022_3347_MOESM5_ESM.tif]

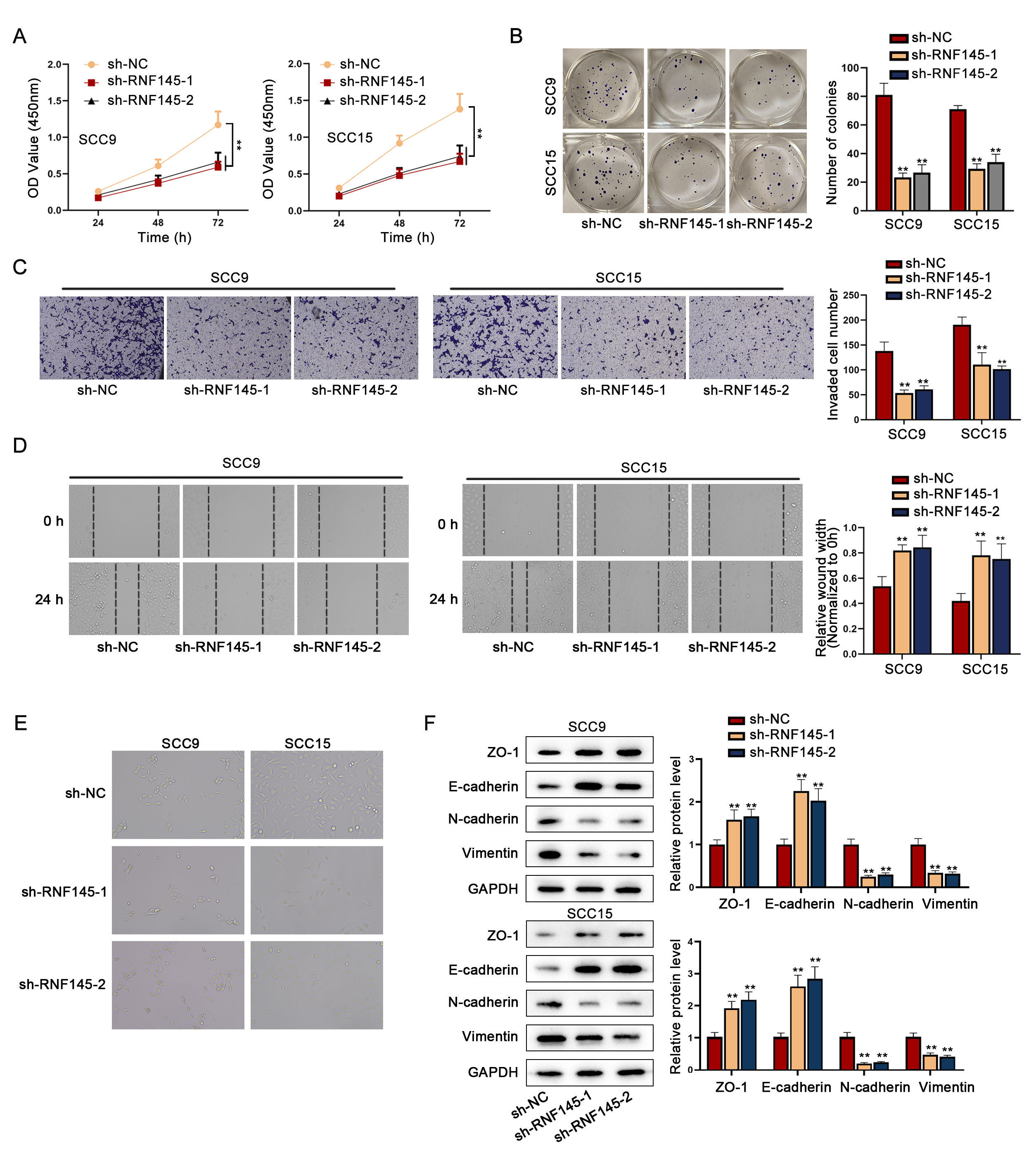

Supplement: Supplementary file 6 — Additional file 6: Figure S6. (A, B) CCK-8 and colony formation assays were applied for measuring cell viability and proliferation when RNF145 was silenced. (C, D) Transwell assay and wound healing assay were utilized to assess cell invasion and migration when RNF145 was inhibited in OSCC cells. (E, F) EMT phenotype was detected in sh-RNF145-1/2-transfected SCC9 and SCC15 cells. **P < 0.01. [file 12967_2022_3347_MOESM6_ESM.tif]

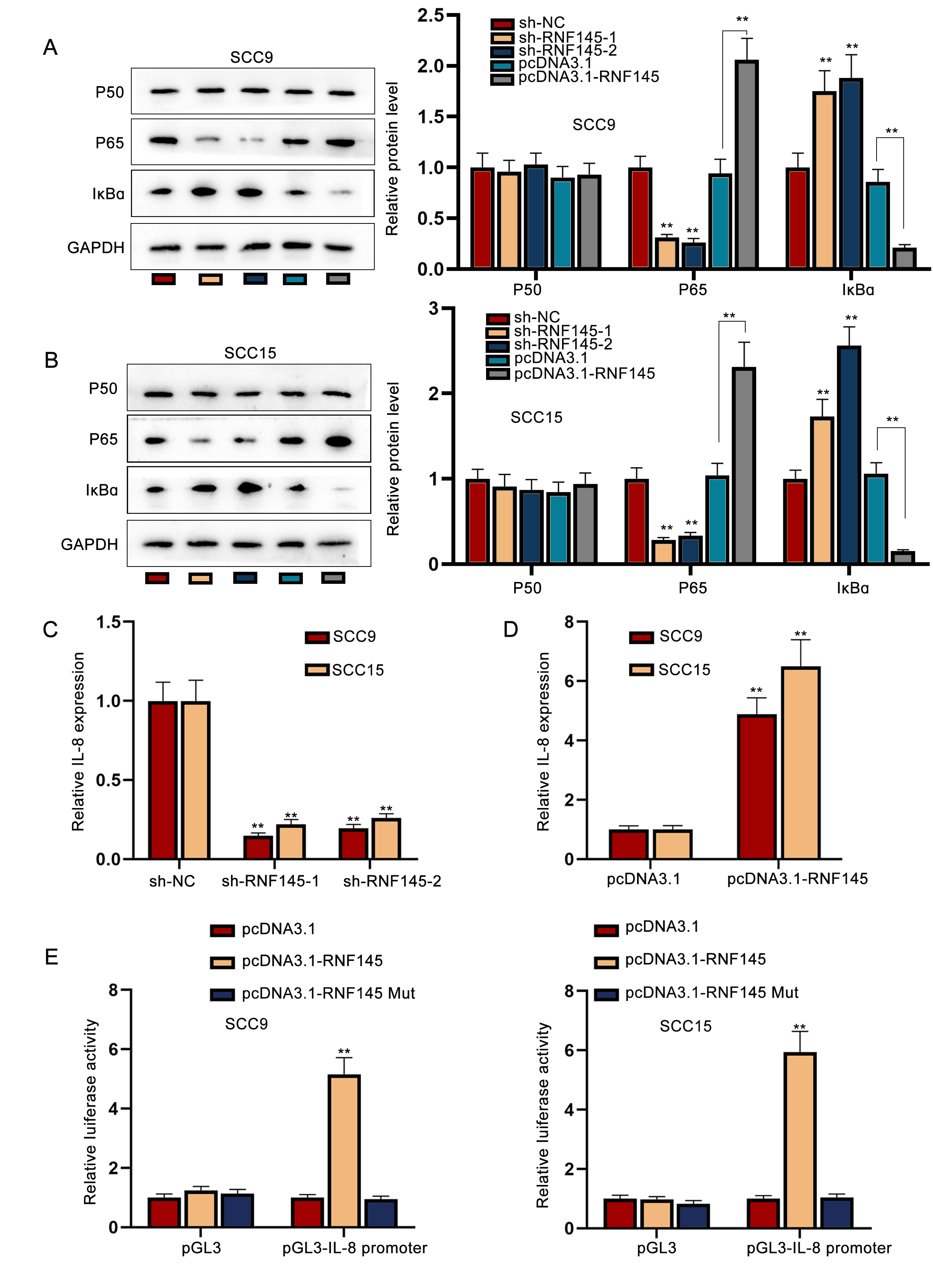

Supplement: Supplementary file 7 — Additional file 7: Figure S7. (A, B) Western blot as well as qRT-PCR was used to detect the expression level of P50, P65 and IκBɑ after RNF145 was silenced or overexpressed in OSCC cells. (C, D) IL-8 expression was detected in OSCC cells after RNF145 was silenced or overexpressed. (E) Luciferase reporter assay was taken to analyze the regulation of IL-8 transcription before and after RNF145 knockdown in OSCC cells. *P < 0.05, **P < 0.01. [file 12967_2022_3347_MOESM7_ESM.tif]
